# Appendiceal Perforation From Muscular Thinning in Limited Cutaneous Systemic Sclerosis: A Case Report

**DOI:** 10.7759/cureus.96196

**Published:** 2025-11-06

**Authors:** Yuki Nakamura, Goshi Fujimoto, Satoshi Matsuda, Hiroshi Kusanagi

**Affiliations:** 1 Gastroenterological Surgery, Kameda Medical Center, Kamogawa, JPN; 2 Pediatric Surgery, Kameda Medical Center, Kamogawa, JPN

**Keywords:** appendix, autoimmune, bowel, colon, fibrosis, necrosis, sclerosis

## Abstract

Systemic sclerosis (SSc) is a multi-system autoimmune disease characterized by vasculopathy and fibrosis. Gastrointestinal involvement is common; however, bowel perforation is rare and carries high morbidity.

We report a 77-year-old woman with limited cutaneous SSc and primary biliary cirrhosis who presented with right lower abdominal pain and cutaneous necrosis. Imaging revealed a retroperitoneal abscess contiguous with the ileocecal region. Surgery demonstrated complete transection of the appendix at its base with preserved serosal color and marked bowel wall fragility, prompting ileocecal and partial transverse colectomies with functional end-to-end anastomoses. Histology revealed muscularis propria thinning and fibrosis extending to the terminal ileum and colon, and non-inflammatory thrombotic occlusion of small- to medium-sized vessels, consistent with SSc-related vasculopathy. Postoperatively, the patient developed anastomotic leakage due to muscular thinning and died on postoperative day 22.

This case illustrates that diffuse intestinal wall fragility in SSc may lead to perforation and anastomotic failure; thus, surgeons should minimize anastomoses and consider stoma creation in such patients.

## Introduction

Systemic sclerosis (SSc) is a chronic autoimmune connective tissue disease characterized by widespread noninflammatory vascular injury, immune dysregulation, and progressive fibrosis of the skin and internal organs. Gastrointestinal (GI) involvement occurs in up to 90% of patients and represents the most frequent internal organ manifestation [1-3]. Pathological changes in the GI tract include vascular damage, fibrosis of the muscularis propria, and enteric neuropathy, all of which collectively result in impaired intestinal motility and structural fragility [4]. Although upper GI symptoms are frequently recognized, lower GI involvement is often underappreciated. Muscular atrophy and fibrosis may manifest in the colon and small intestine such as pseudo-obstruction, and, in rare but serious cases, spontaneous perforation [5-7]. Notably, bowel perforations in SSc are often associated with poor outcomes owing to delayed recognition and impaired healing capacity of the diseased intestinal wall.

Here, we report a rare case of retroperitoneal appendiceal perforation complicated by cutaneous necrosis and subsequent postoperative anastomotic leakage in a patient with systemic sclerosis (SSc). Although bowel perforation has been described in patients with SSc, what distinguishes this case is the fatal postoperative anastomotic leakage, demonstrating the diffuse fragility of the gastrointestinal wall in limited cutaneous SSc. This case highlights the importance of early recognition and careful surgical strategies in the management of patients with SSc.

## Case presentation

A 77-year-old woman with a history of limited cutaneous systemic sclerosis (lcSSc) and primary biliary cirrhosis (PBC) since early adulthood presented for evaluation. Her medical history included hypertension, dyslipidemia, and gastroesophageal reflux disease (GERD). She had no prior exposure to corticosteroids or other immunosuppressive therapy. Her body mass index (BMI) was 16 kg/m^2^. Four months prior to admission, she developed right lower abdominal pain (Table 1). Her symptoms were mild (Visual Analog Scale (VAS) score 2), and inflammatory markers were only slightly elevated; therefore, no imaging was performed, and she was monitored conservatively. Subsequently, one month prior to admission, she experienced progressive difficulty with movement. Three days before admission, skin necrosis appeared near the right anterior superior iliac spine. Finally, the combination of necrosis and progressive weakness prompted presentation to the emergency department (ED). She was febrile but hemodynamically stable and had localized right lower abdominal pain without peritoneal signs. On examination, a 5 cm necrotic lesion with surrounding warmth and tenderness was observed. Laboratory tests revealed leukocytosis (27,800/µL; reference interval 3,300-8,600/µL), anemia (hemoglobin 6.8 g/dL; reference interval 13.7-16.8 g/dL), elevated C-reactive protein (15.49 mg/dL; reference interval <0.14 mg/dL), and impaired coagulation (prothrombin activity, 64.1%; reference interval 80-120%). Child-Pugh score was 7 (Grade B), which is associated with an approximately 80% one-year survival rate in patients with liver cirrhosis.

**Table 1:** Clinical timeline of patient events before and after admission (–) indicates time before admission, (+) indicates time after admission POD: postoperative day, VAS: Visual Analogue Scale; NA: not applicable

Time before/after admission	Event	Notes/POD
(-) Four months	Right lower abdominal pain	Mild (VAS 2), mild inflammation, no imaging
(-) One month	Progressive difficulty with movement	NA
(-) Three days	Skin necrosis appeared near the right anterior superior iliac spine	NA
Admission day	Bedside drainage	NA
(+) One day	Fecal drainage was performed	NA
(+) Two days	Colonoscopy	Leakage into retroperitoneum
(+) Five days	Surgery was performed	NA
(+) 14 days	Resumed oral intake	POD 9
(+) 20 days	Anastomotic leakage	POD 15
(+) 27 days	Died	NA

**Figure 1:**
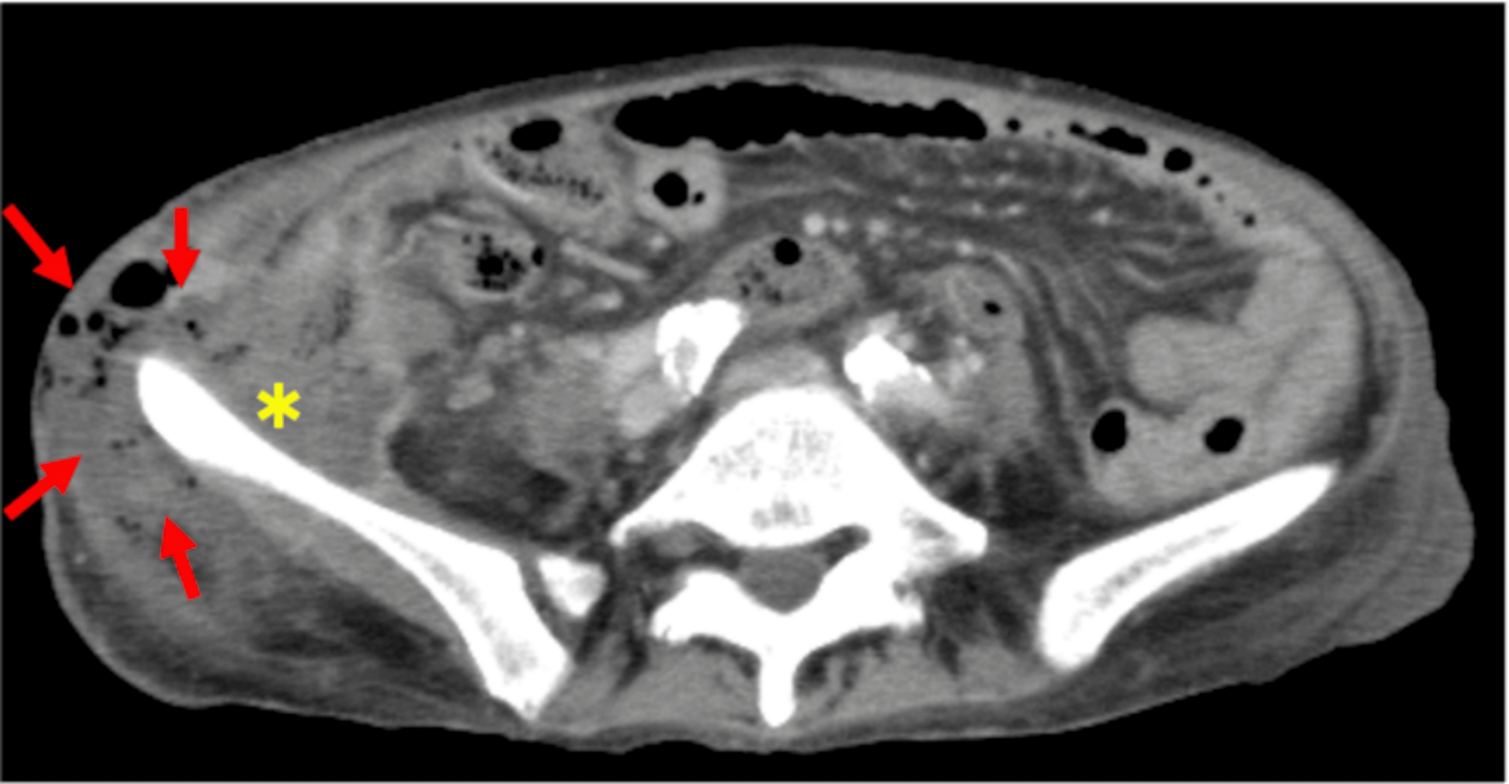
Computed tomography image at initial presentation An abscess is observed on the anterior aspect of the ilium (*) with a fistulous tract extending to the skin (→)

Contrast-enhanced computed tomography (CT) revealed an abscess in the right iliac fossa with gas, contiguous with necrotic skin; however, no intraperitoneal free air was observed (Figure 1).

**Figure 2:**
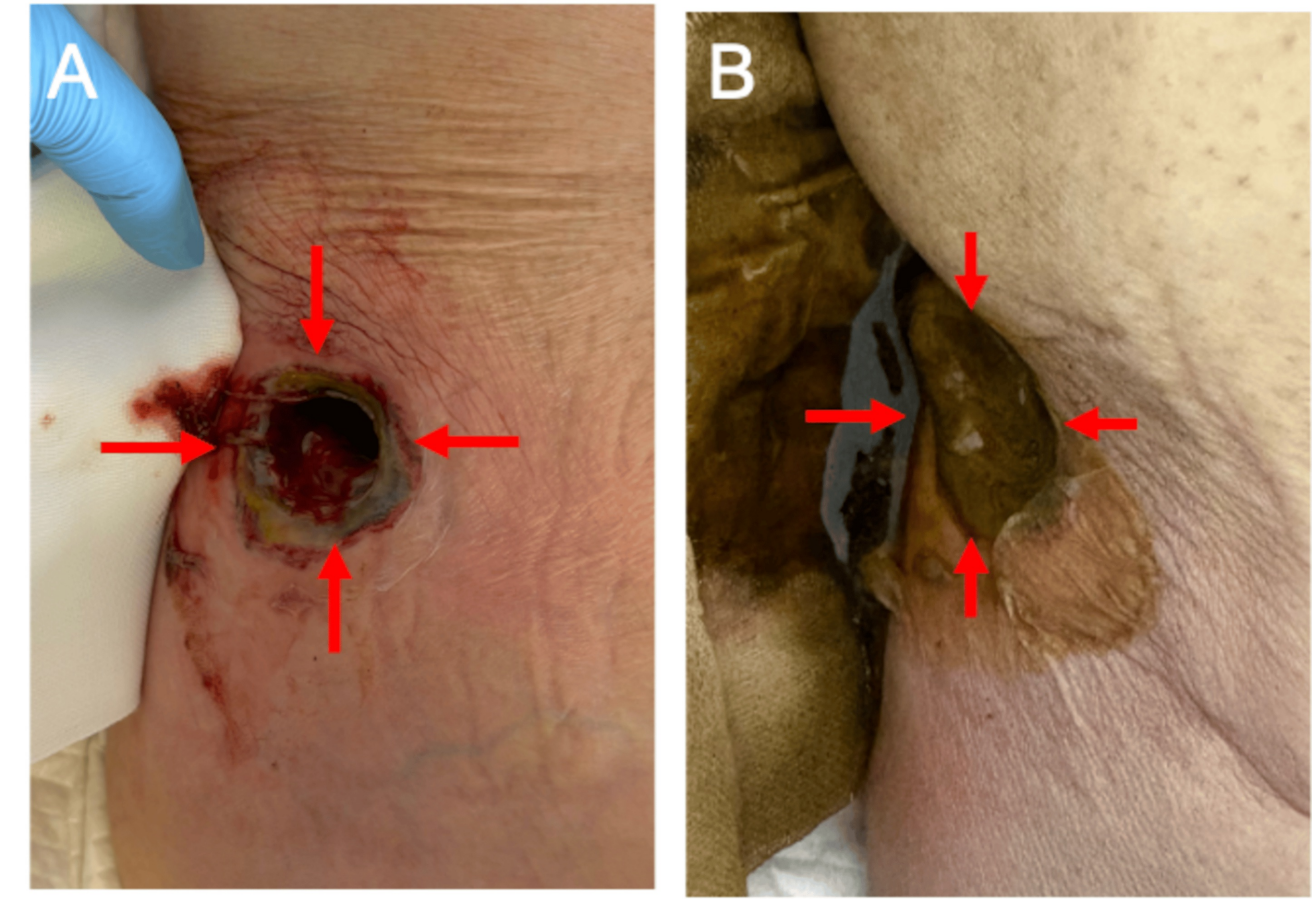
Skin fistula through the abscess cavity (A) Bedside incision of the necrotic skin released old hematoma in the abscess cavity (→) (B) On the following day, stool was drained from the fistula (→)

A bedside incision of the necrotic skin released blood-stained, purulent fluid, and a drain was placed in the abscess cavity (Figure 2A). Cultures grew *Escherichia coli*. Fecal drainage was performed on the following day (Figure 2B).

**Figure 3:**
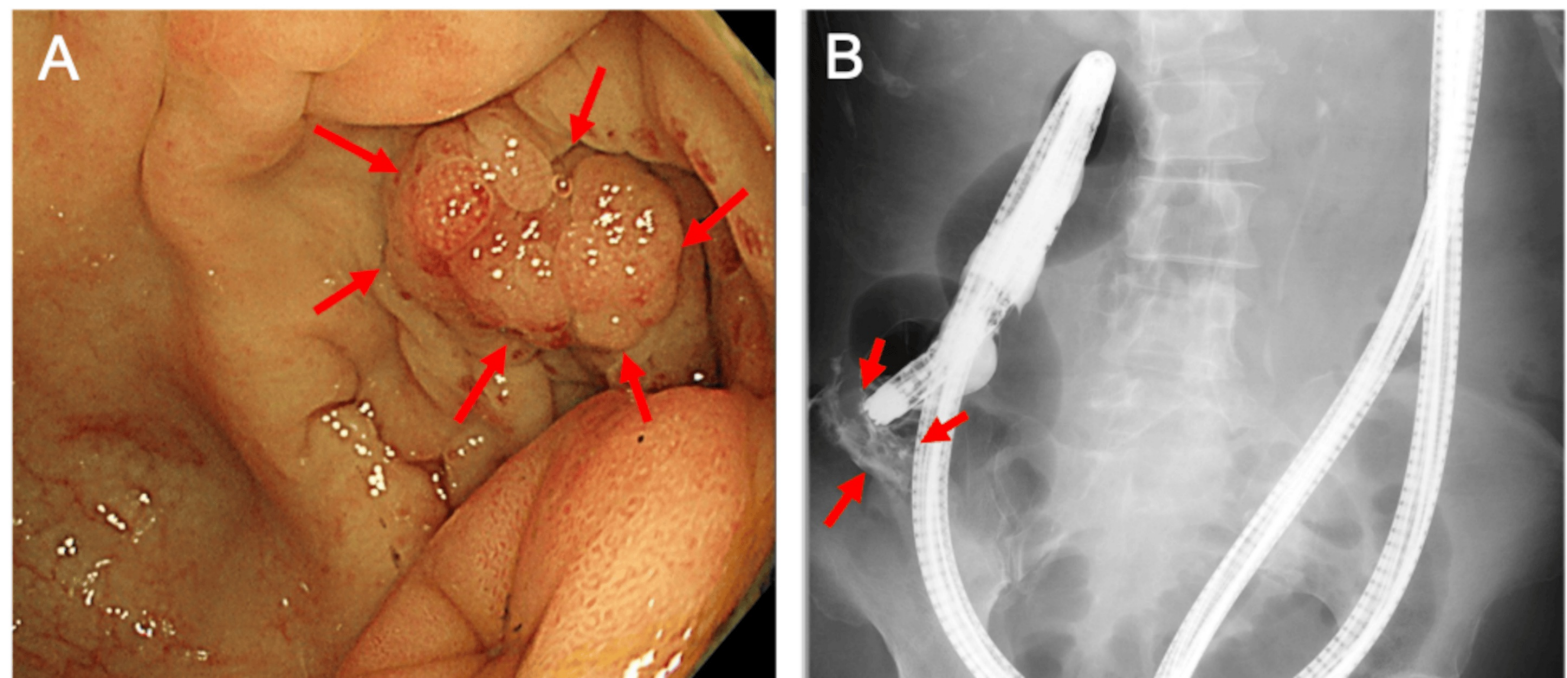
Fluoroscopic-aided colonoscopy (A) No diverticula or intraluminal cancer was observed, although extramural compression was suspected around the appendiceal orifice (→) (B) Infusion of the contrast visualized the fistula to the skin (→)

Colonoscopy revealed an extrinsic compressive lesion at the appendiceal orifice (Figure 3A), and contrast injection revealed leakage into the retroperitoneum (Figure 3B).

**Figure 4:**
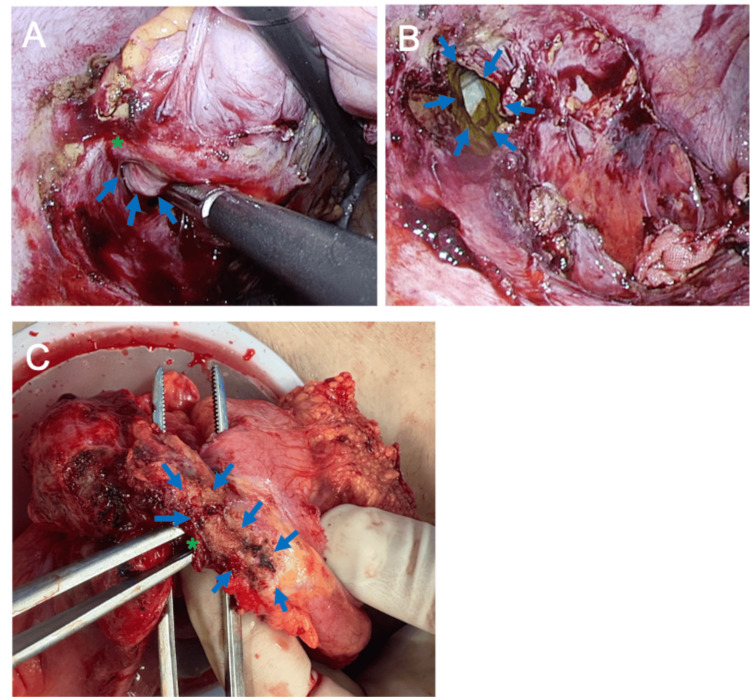
Intraoperative findings (A) The appendix was ruptured at its base (*), forming a connection with the abscess cavity (→) (B) The abscess had formed a fistula to the skin (→) (C) The transverse colon next to the abscess cavity was slightly torn (*), and the wall of the transverse colon appeared fragile after dissection (→)

Given the normal mucosal findings without ulceration, Crohn’s disease was considered unlikely, and diverticulitis was excluded because no diverticula were present. The cecum appeared indistinct on CT, and the unusual combination of a retroperitoneal abscess and cutaneous necrosis raised concern for an underlying malignancy or submucosal process. Based on the collective findings, retroperitoneal appendiceal perforation was considered the most likely diagnosis, although tumor-related perforation could not be completely ruled out preoperatively. Surgery revealed dense adhesions between the cecum and the transverse colon, with an abscess cavity extending to the skin fistula. The appendix was completely transected at its base and communicated with the abscess cavity, although its serosal color appeared grossly preserved (Figures 4A, 4B). An ileocecal resection was performed. During dissection, the transverse colon adherent to the abscess cavity was slightly torn. Although bowel injury was limited to a small perforation owing to the fragility of the transverse colon wall, a partial transverse colectomy was added (Figure 4C). Preoperatively, no radiologic evidence suggested bowel wall thinning or ischemia attributable to systemic sclerosis, and dense adhesions around the hepatic flexure raised concern for additional colon injury with further mobilization. Given these considerations and the absence of intraperitoneal contamination, two functional end-to-end anastomoses (FEEA), a technique joining bowel ends, were selected over stoma creation.

**Figure 5:**
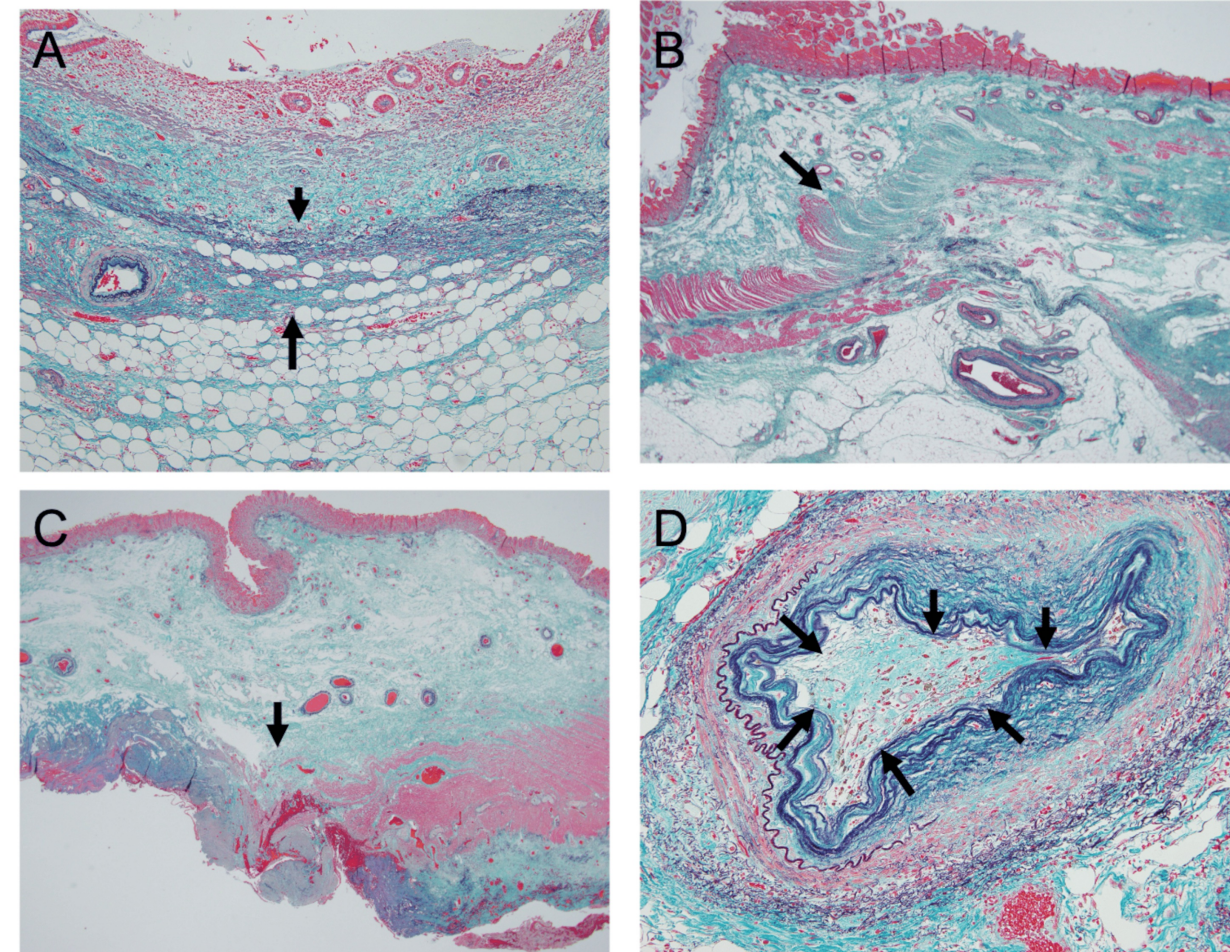
Pathological findings All specimens were stained with Elastica-Goldner; magnifications were ×100, ×2, ×2, and ×400, respectively (A) Appendix wall, showing loss of the muscular layer, which is replaced by fibrosis (→) (B) Terminal ileum wall, with fibrosis replacing the muscular layers (both inner circular and outer longitudinal) from the arrow (→) (C) Ascending colon wall, with loss of the muscular layer (→) (D) Small artery with complete fibrotic occlusion due to organized thrombus (→)

Histological examination revealed a perforated appendicitis with abscess formation. No significant necrosis of the appendiceal wall was observed, suggesting that the perforation was not due to localized gangrenous appendicitis. Notably, diffuse thinning and fibrosis of the muscularis propria were present not only in the appendix but also in the terminal ileum and ascending colon. Non-inflammatory thrombotic occlusion of small- to medium-sized vessels was also observed, which was consistent with systemic sclerosis-related vasculopathy (Figure 5). Diverticula were not observed.

**Figure 6:**
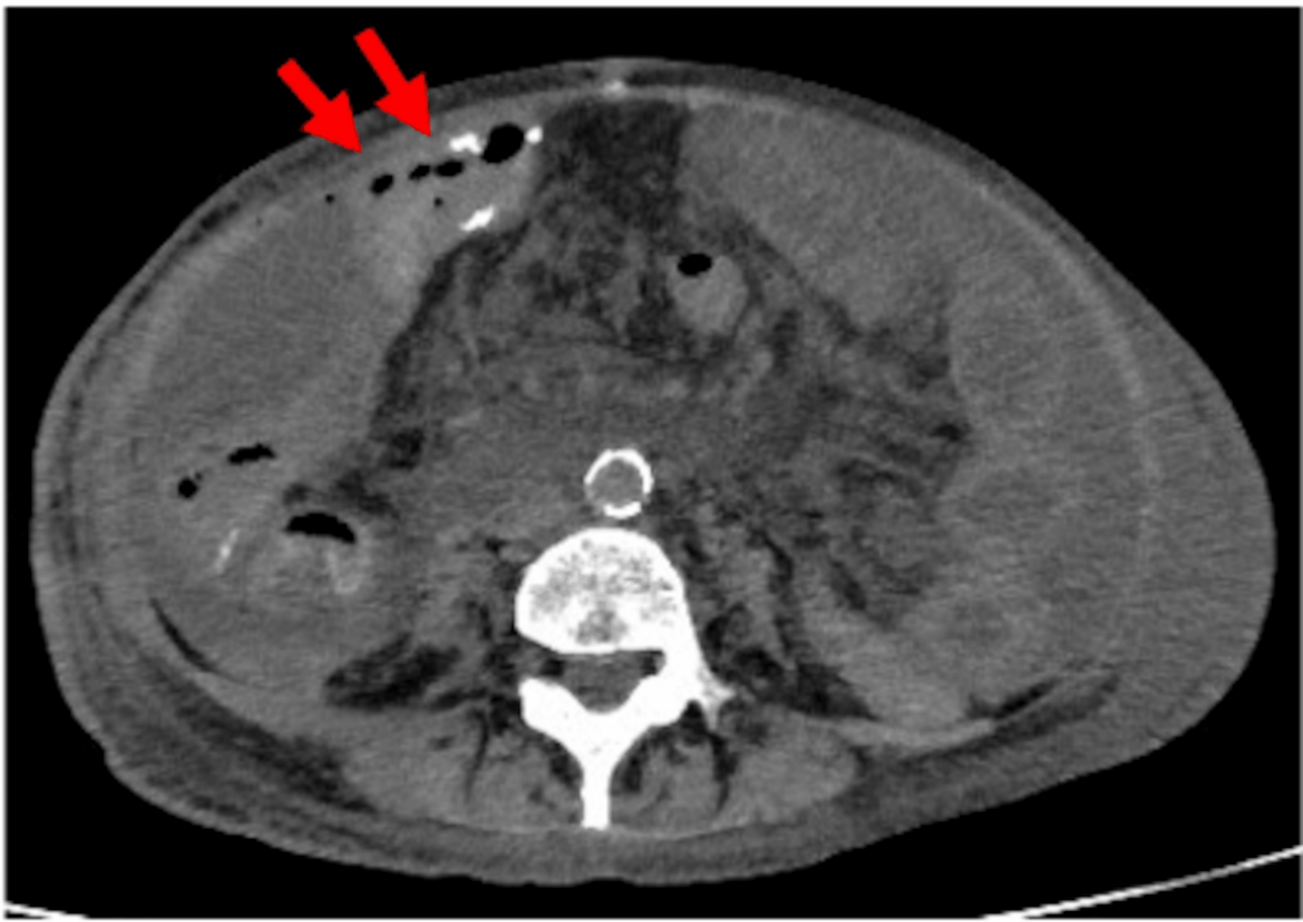
Computed tomography image on postoperative day 15 Free air was observed around the transverse colonic anastomosis, leading to the diagnosis of anastomotic leakage (→)

The patient resumed oral intake on postoperative day (POD) 9. However, on POD 15, she developed worsening abdominal pain, and CT confirmed an anastomotic leakage of the transverse colon (Figure 6). Although the creation of an ileostomy was proposed, she declined further surgery. Despite supportive care, the patient died on POD 22.

## Discussion

SSc is a chronic autoimmune disease characterized by vasculopathy and progressive fibrosis of connective tissues. Gastrointestinal involvement is common, affecting up to 90% of patients, and may include dysphagia, GERD, small intestinal bacterial overgrowth (SIBO), pseudo-obstruction, pneumatosis cystoides intestinalis, and, rarely, perforation [1-7]. Pathological features typically include subintimal fibrosis of the arteries, smooth muscle atrophy, and loss of enteric nerves, which collectively impair motility and weaken the bowel wall [7].

Colonic involvement has been diagnosed radiologically or endoscopically in 20-50% of patients with SSc, often without overt symptoms. Constipation due to colonic dysmotility or muscular atrophy is relatively common [3]. Although colonic perforation is rare, it represents a serious complication, making early recognition and intervention essential [8]. Previous reports have described patients in whom radiological findings such as pneumatosis intestinalis or bowel wall thinning enabled timely management before perforation occurred [9]. However, in most cases, perforation was only identified after the onset of peritoneal signs, leading to CT imaging and subsequent surgery [3,10,11]. Considering the chronic fragility of the gastrointestinal wall in SSc, abdominal pain should prompt clinicians to consider early imaging and additional evaluation.

Gastrointestinal perforations in autoimmune diseases provide an opportunity to contrast the pathogenic mechanisms underlying systemic lupus erythematosus (SLE) and SSc. In SLE, bowel perforation usually results from lupus enteritis, in which immune complex-mediated inflammation of the mesenteric vessels leads to ischemia and necrosis [12]. Clinically, SLE-associated perforations tend to occur acutely during disease flares and may be multifocal [13]. In contrast, SSc-related perforations are usually non-inflammatory and develop insidiously due to chronic ischemia from intimal fibrosis, smooth muscle atrophy, and non-inflammatory thrombotic occlusion [5]. Such comparisons are clinically important because the underlying differences in pathogenic mechanisms may affect surgical choices and postoperative care.

In this case, histological examination demonstrated diffuse thinning and fibrosis of the muscularis propria in both the appendix and the adjacent ileal and colonic segments. The absence of marked appendiceal necrosis indicates that the perforation was not simply the result of localized inflammatory destruction, but rather reflected diffuse bowel wall fragility associated with systemic sclerosis. The presence of non-inflammatory thrombotic occlusion in small- and medium-sized vessels further supports systemic sclerosis as the underlying etiology, rather than a simple localized appendiceal disease. In SSc patients, vascular damage leads to impaired tissue perfusion and increased risk of ischemia, while neuronal damage causes intestinal hypomotility, resulting in stasis of luminal contents, bacterial overgrowth, distension, and increased traction forces. Concurrently, muscular damage weakens the bowel wall’s resistance to tension. The chronic interplay of these three processes, occurring in parallel, culminates in silent ischemic weakening of the intestinal wall. In this patient, vascular and muscular damage were diffusely distributed, suggesting that the bowel had reached an advanced stage of chronic SSc-related weakening.

Bowel fragility in patients with systemic sclerosis has important implications for surgical management. In our case, multiple anastomoses were performed using the FEEA technique. However, anastomotic leakage developed on POD 15. We believe that this complication was primarily due to a severely weakened intestinal wall. Previous reports emphasized the importance of recognizing intestinal wall fragility during invasive procedures (Table 2) [5,8-11,14].

**Table 2:** Summary of reported intestinal perforation cases in systemic sclerosis This table compares previously reported cases of systemic sclerosis–related gastrointestinal perforation with the present case, focusing on patient demographics, perforation site, clinical presentation, histopathology, surgical approach, and outcomes.

Author/year	Age/gender	Perforation site	Presentation	Histology findings	Surgical approach	Stoma	Outcome
Stupalkowska et al. (2020) [8]	49, female	Sigmoid colon	Intraperitoneal	Muscular thinning, fibrosis	Resection + open abdominal management	No	Died (29 hours)
Guan et al. (2018) [9]	78, female	Ascending colon	Intraperitoneal	Transmural ischemic necrosis and fibrosis	Subtotal colectomy	Yes	Died
Jayson et al. (1972) [10]	35, female	Rectosigmoid junction	Intraperitoneal	Muscular thinning, fibrosis, vasculopathy	Sigmoidectomy	Yes	Survived
Goodwin et al. (2023) [11]	60, female	Descending colon	Intraperitoneal	Not described	Partial left hemicolectomy	Yes	Survived
Oiwa et al. (2005) [14]	50, male	Jejunum (afferent loop)	Intraperitoneal	Muscular thinning without fibrosis	Resection + fistulization	No	Died (2 months)
Present case	77, female	Appendix	Retroperitoneal	Muscular thinning, fibrosis, vasculopathy	Ileocecal resection + partial transverse colectomy	No	Died (27 days)

Most reported cases involved intraperitoneal colonic perforation associated with muscular thinning or ischemic necrosis. In contrast, the present case uniquely demonstrated retroperitoneal appendiceal perforation. While all previous cases either underwent or planned for stoma creation or anticipated fistula formation [9-11,14], the present case was managed without stoma formation. In previous reports, stoma creation was necessary due to intraperitoneal contamination, leaving no opportunity to consider intestinal wall fragility for safe anastomosis. In contrast, in our case, contamination was confined to the retroperitoneal space, requiring careful assessment of whether a safe anastomosis could be performed. In our case, primary anastomosis was selected for three reasons: preoperative imaging revealed no SSc-related bowel fragility, dense adhesions limited further mobilization, and there was no intraperitoneal contamination. Retrospectively, however, in patients with suspected SSc-related bowel fragility, single anastomosis or diversion should be prioritized. In addition, our patient had primary biliary cirrhosis (PBC), which has been reported to coexist with limited cutaneous SSc in approximately 2-3% of cases [15]. While PBC itself does not directly cause intestinal fragility, it may contribute to surgical risk through impaired hepatic function and coagulation abnormalities, as evident in our case with anemia and reduced prothrombin time activity. Additionally, the patient’s underweight status (BMI 16) likely impaired anastomotic healing capacity.

Another unique aspect of this case was the retroperitoneal extension of the appendiceal perforation, which led to atypical clinical features. Unlike classical appendicitis, which presents with peritoneal irritation following intraperitoneal rupture, retroperitoneal perforation may cause subtle or delayed symptoms. The initial manifestation of cutaneous necrosis further complicated timely diagnosis; however, as in previous reports, fluoroscopy-assisted endoscopy contributed to establishing the diagnosis [16]. In addition, although the necrosis was primarily associated with retroperitoneal infection, it is plausible that the underlying microvascular ischemia due to SSc contributed to impaired tissue perfusion and exacerbated skin breakdown. This overlap between infection-related tissue injury and disease-related microvasculopathy may have further obscured the clinical presentation.

The main limitation of this case is the lack of pathological evaluation of the transverse colon wall responsible for the anastomotic leakage, since a pathological autopsy was refused by the patient and her family. The absence of transverse colon pathology limits confirmation, though similar fragility is plausible given the diffuse findings.

This case demonstrates that patients with SSc presenting with abdominal pain require a high index of suspicion and early imaging, even when symptoms are mild or atypical.

## Conclusions

This case demonstrates that systemic sclerosis may predispose patients to intestinal perforation and postoperative anastomotic leakage due to diffuse intestinal wall fragility. Clinicians should maintain a low threshold for advanced imaging in these patients and adopt careful surgical strategies, including minimizing the number of anastomoses and considering diverting stoma creation when perforation is encountered. Multidisciplinary management involving rheumatology, surgery, radiology, and other relevant specialties should also be considered.
